# A Rational Approach for the Identification of Non-Hydroxamate HDAC6-Selective Inhibitors

**DOI:** 10.1038/srep29086

**Published:** 2016-07-12

**Authors:** Laura Goracci, Nathalie Deschamps, Giuseppe Marco Randazzo, Charlotte Petit, Carolina Dos Santos Passos, Pierre-Alain Carrupt, Claudia Simões-Pires, Alessandra Nurisso

**Affiliations:** 1 School of Pharmaceutical Sciences, University of Geneva, University of Lausanne Quai Ernest-Ansermet, 30, CH-1211, Geneva 4, Switzerland; 2 Laboratory for Cheminformatics and Molecular Modeling, Department of Chemistry, Biology and Biotechnology, University of Perugia, Via Elce di Sotto, 8, 06123 Perugia, Italy; 3 Département de Biochimie, Université de Montréal, H3C 3J7 Montréal, Québec, Canada

## Abstract

The human histone deacetylase isoform 6 (HDAC6) has been demonstrated to play a major role in cell motility and aggresome formation, being interesting for the treatment of multiple tumour types and neurodegenerative conditions. Currently, most HDAC inhibitors in preclinical or clinical evaluations are non-selective inhibitors, characterised by a hydroxamate zinc-binding group (ZBG) showing off-target effects and mutagenicity. The identification of selective HDAC6 inhibitors with novel chemical properties has not been successful yet, also because of the absence of crystallographic information that makes the rational design of HDAC6 selective inhibitors difficult. Using HDAC inhibitory data retrieved from the ChEMBL database and ligand-based computational strategies, we identified 8 original new non-hydroxamate HDAC6 inhibitors from the SPECS database, with activity in the low μM range. The most potent and selective compound, bearing a hydrazide ZBG, was shown to increase tubulin acetylation in human cells. No effects on histone H4 acetylation were observed. To the best of our knowledge, this is the first report of an HDAC6 selective inhibitor bearing a hydrazide ZBG. Its capability to passively cross the blood-brain barrier (BBB), as observed through PAMPA assays, and its low cytotoxicity *in vitro,* suggested its potential for drug development.

Histone deacetylases (HDACs) are part of the epigenetic machinery. Within histone acetyltransferases, they are responsible for controlling the acetylation status of histones, regulating chromatin condensation and gene expression. Over the past decades, HDACs have emerged as promising therapeutical targets for cancer and neurodegenerative diseases because of their modulation in hypoacetylated conditions typical of such disorders^1^,^2^,^3^. HDAC enzymes may be classified in four classes based on phylogenetics: class I (HDAC1-3, 8), class II (class IIa: HDAC4, 5, 7, 9; and class IIb: HDAC6, 10), class III (sirtuins SIRT1-7), and class IV (HDAC11). HDACs classes I, II, and IV are zinc-dependent enzymes, whereas class III HDACs are NAD^+^-dependent proteins^2^. All zinc-dependent isoforms share a catalytic site with similar structural properties, and are either nuclear or shuttle between the nucleus and the cytoplasm. HDAC6 is a mainly cytosolic isoform that targets non-histone substrates, such as α-tubulin, HSP90, and cortactin controlling microtubule-dependent cell motility and degradation of misfolded proteins through the aggresome pathway. These properties make HDAC6 a target of interest because of its potential role in cancer and neurodegenerative disorders^3^,^4^,^5^,^6^,^7^,^8^.

Considerable efforts have been made to develop HDAC inhibitors, and some of them have even reached the market as antitumor drugs, such as Vorinostat (SAHA), Romidepsin (FK228, a prodrug), Belinostat (PXD-101), and Panabinostat (LBH-589, Farydak, www.fda.gov)^9^,^10^. All of these non-selective HDAC inhibitors share the prototypical pharmacophoric scheme for HDAC inhibition, consisting of a zinc binding group (ZBG), a hydrophobic linker or spacer to fit the catalytic site channel, and a cap group targeting the channel rim (Fig. 1A)^11^. According to crystallographic and biological information, the cap group was identified as being mainly responsible for HDAC isoform selectivity^12^,^13^,^14^,^15^, a hypothesis that has recently been questioned for HDAC6^16^,^17^.

There are a limited number of studies on the modulation of ZBG. Indeed, the study of this modulation is quite challenging because of the high homology characterising the metal-dependent catalytic core of HDAC proteins. Moreover, current computational methodologies for modelling zinc ion properties are limited, which makes virtual screening results difficult to evaluate. The zinc ion can be defined as a borderline acid, with intermediate properties between hard and soft Lewis acids. Its coordination geometry and interaction strength within heteroatoms are very difficult to retrieve *in silico*^18^,^19^. The ZBG is normally characterised by a hydroxamic acid (HA) moiety. Unfortunately, HA is known to suffer from significant off-target effects, including interaction with the hERG cardiac potassium channel and mutagenic effects. In terms of pharmacokinetics, HA displays low bioavailability and a short intravenous half-life. Moreover, in cells, HA can be easily hydrolysed into carboxylic acid, and metabolised *via* sulphation and glucuronidation^20^,^21^,^22^. In addition to HA, carboxylates, anilides and thiols have been considered as alternative ZBGs able to inhibit HDAC enzymes^23^,^24^,^25^,^26^. Therefore, ZBG modulation is of great interest in the search for selective and less toxic HDAC inhibitors.

Structure-based strategies have been widely adopted in the past for the design of class I-II HDAC inhibitors due to abundant crystallographic data^27^,^28^,^29^,^30^,^31^,^32^,^33^. To date, no crystallographic information is available for the HDAC6 catalytic pocket, limiting the rational design of new selective inhibitors. Tubastatin A and other selective HDAC6 inhibitors have been discovered through screening strategies coupled to structure-activity relationship (SAR) and computational interaction studies using HDAC6 homology models^34^,^35^,^36^,^37^,^38^.

To the best of our knowledge, pharmacophore- or ligand-based approaches have never been considered in the discovery of new HDAC6-selective inhibitors. Thus, the aim of the present study is to use information from ligands of known potency and selectivity to carry out a virtual screening campaign able to identify novel and selective HDAC6 inhibitors, ideally possessing an original ZBG. The general approach is summarised in Fig. 1B.

## Results

### Generation of a pharmacophore model for HDAC6 catalytic inhibitors

**Figure 2:**
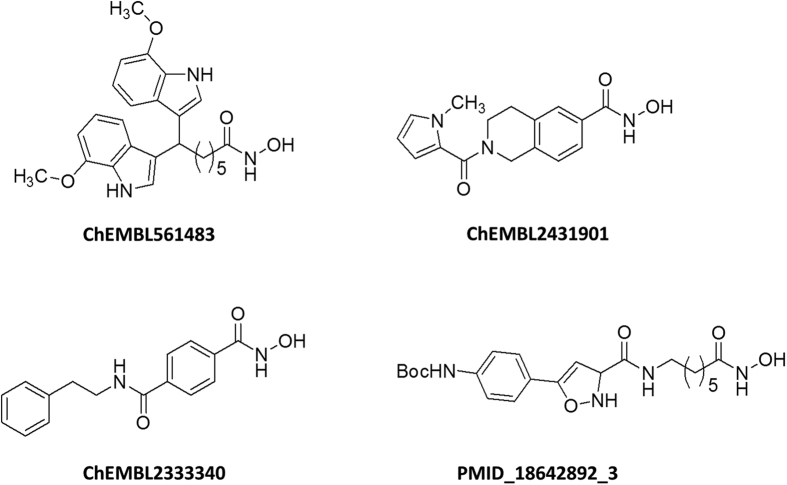
Chemical structures of compounds selected for pharmacophore generation. The structure of the most potent HDAC6 inhibitor (PMID_18642892_3) reported in the literature is also presented.

**Figure 3:**
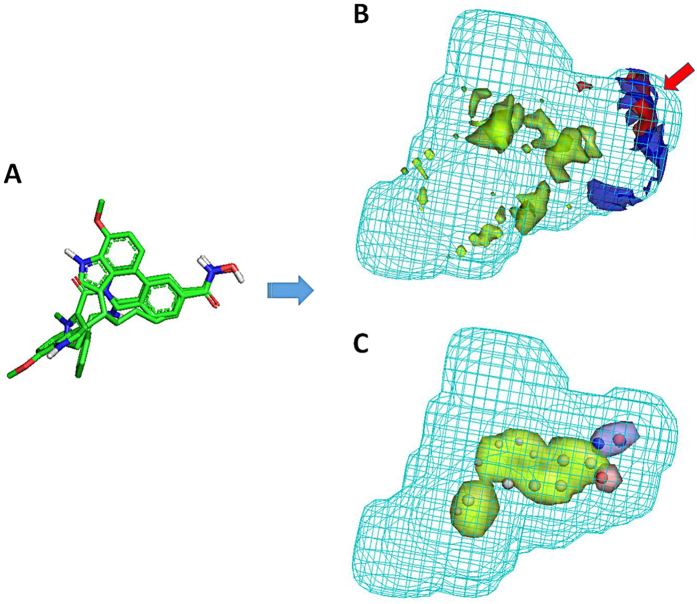
Pharmacophore model for selective HDAC6 inhibitors according to FLAPpharm. (**A**) Alignment obtained for the three compounds. (**B**) Pharmacophore obtained upon alignment in terms of common pharmacophoric interaction fields (PIFs). The hydrogen-bond donor constraint used for screening is indicated by a red arrow. (**C**) Pharmacophore obtained upon alignment in terms of common pharmacophoric points at the centroid of pseudoPIFs. The most relevant common pharmacophoric points at the centroid of the pseudoPIFs are also highlighted. For both pharmacophore depictions (**B**, **C**), the green areas represent the hydrophobic moieties, the blue areas represent the H-bond donor regions, the red areas represent the H-bond acceptor regions, and the cyan wireframe surface defines the shape of the pharmacophore.

The ChEMBL compound collection was used as a source of HDAC inhibitory information. This dataset was conceived with the final aim of generating a discriminative HDAC6 pharmacophoric model. For this reason, data on HDAC isoforms other than HDAC6 were also collected: HDAC2 and 8, representing class I HDAC enzymes; HDAC4, representing class IIa HDACs. The FLAPpharm algorithm^39^ was then used with the aim of building a robust pharmacophore model for HDAC6 catalytic inhibitors. This approach has been successfully used in the past for constructing a discriminating toxicophore model for phospholipidosis (PLD) inducers, for differentiating adenosine receptor subtypes, and recently for discovering inhibitors of a novel drug/proton antiporter^40^,^41^,^42^. In order to generate a pharmacophore by using the FLAPpharm approach, a minimum of three molecules is required^39^. These molecules were taken from our ChEMBL dataset that here we called HDAC ChEMBL dataset (Table S1). Several attempts were made to select the three best molecules for pharmacophore generation, considering their activity, selectivity, and, whether possible, structural diversity. Compounds having IC_50_ < 50 nM and selectivity index (SI) > 100 with respect to HDAC6 were selected for a first pharmacophore building attempt. In order to model a robust pharmacophore model based on drug-like compounds, a MW filter was set at 600 Da, also excluding macrolide-like structures. Unfortunately, with these cut-off parameters, only one compound matched the search criteria (ChEMBL333340). Cut-off parameters were therefore modified to IC_50_ < 100 nM, SI > 50, and MW < 600 Da (macrolides excluded, Tables S2, S3), allowing the selection of three compounds (Fig. 2). These compounds were automatically aligned by FLAPpharm, and their best alignment in terms of S-score value^39^ (Fig. 3A) was used to define the pharmacophore. Based on this approach, the pharmacophore is described as a ‘pseudomolecule’, which is composed of common pharmacophoric interaction fields (PIFs), (Fig. 3B) and common atom-centred pseudopharmacophoric fields (pseudoPIFs) (Fig. 3C). The pharmacophore obtained for HDAC6 selective inhibitors is composed of an extended hydrophobic core (green region and points, Fig. 3B). The H-bond donor region (blue region and points, Fig. 3B), and the H-bond acceptor (red region and points) portion accommodate the HA moiety common to the three molecules (Fig. 2).

### Validation of the HDAC6 pharmacophore model

The discriminatory power of the HDAC6 pharmacophore model was evaluated through the screening of a subset of molecules extracted from the HDAC ChEMBL dataset. Compounds within IC_50_ ≤ 10 μM were considered active, whereas compounds with IC_50_ ≥ 100 μM were considered inactive. The validation set comprised 84 compounds (66 active molecules; 18 non-active molecules), as reported in Table S2. All the ligands of the validation set were screened using the best pharmacophoric model as a template, in the PIFs mode (Fig. 3B). Indeed, the use of common GRID fields among the aligned molecules rather than the common atomic coordinates or the pseudoPIFs (Fig. 3C) for compound retrieval allows to investigate a broader chemical space. As the majority of HDAC6 inhibitors bind to the zinc ion through an HA group, we decided to add a hydrogen bond donor (HBD) constraint to the pharmacophore to guide the recognition of potential ZBG.

The ROC curves for each one of the 19 FLAP descriptors^39^ were plotted to evaluate the screening results. The best AUC value was obtained when considering the N1*O descriptor (related to a combination of HBD and HBA chemical features), indicating a good discrimination between active and non-active HDAC6 inhibitors (Fig. S1A). Although this result highlights the favourable effect of the HBD constraint used to define a local similarity, in FLAP it is important to check that an acceptable global similarity with the pharmacophore is maintained, to not over-estimate the constraint effect. Indeed, such positive results with the N1*O descriptor were quite expected as none of the 18 non-active compounds contained the HA moiety (Table S2). In this case, a ROC curve with a favourable AUC of 0.88 was also obtained when the more general descriptor Glob-Prod was used to rank the screened results. Taking into account that Glob-Prod descriptor proved to be highly performant in other pharmacophore-based virtual screening (PBVS) campaigns^40^,^41^,^42^, this descriptor was used in this study to rank virtual screening results obtained using the pharmacophore as a template. Indeed, this choice allows evaluating the screening both by local similarity (driven by the use of HBD constraint), and by global similarity (driven by the use of the Glob-Prod descriptor). Finally, it is noteworthy that the pharmacophore was able to retrieve also macrolide-containing HA as active compounds.

### Pharmacophore-based virtual screening (PBVS)

The best way to understand the reliability of a pharmacophore model consists in its use as a template for virtual screening, testing the best hits *in vitro*. In this study, the SPECS database (www.specs.net, database of 10 mg commercially available compounds) was screened considering the HDAC6 pharmacophore as a template. As a first step, a pre-filtering run was performed using the FLAP procedure in the bit-string mode: a similarity score was assigned based on the similarity of quadruplets built on atomic coordinates, without generating the GRID fields. The MW filter (MW < 600 Da) and HBD constraints were applied, obtaining a total of 203,891 compounds to be screened. Twenty-five conformers were generated for each SPECS compound. As a result of this first virtual screening, compounds displaying a Glob-Sum higher than 0.25 were selected (1,791 compounds), and used to generate a GRID fields-based FLAP database for a more accurate similarity evaluation. Indeed, in the prefiltering run, the Glob-Prod descriptor is not available in FLAP, and the Glob-Sum is the descriptor to evaluate the global similarity. A second PBVS was then performed. The accuracy of the FLAPpharm algorithm was set to “normal” and the HBD constraint again applied. Virtual screening results were ranked by Glob-Prod, and the 200 top-ranked compounds were selected for further investigation (Table S4).

### Ligand-based virtual screening (LBVS) using the most potent HDAC6 inhibitor reported in the literature

During the construction of the HDAC ChEMBL dataset, we realised the existence of an HDAC6-selective isoxazole-containing inhibitor with a picomolar activity, named as compound **7** by Kozikowski *et al.*^37^. This compound (PMID_18642892_3 in Fig. 2), was not included in our HDAC ChEMBL dataset, since it has never been tested for HDAC4 inhibition. We believed that this highly potent compound could be used as a template for a novel LBVS campaign, to be performed in parallel to PBVS on the SPECS database (www.specs.net, database of 10 mg commercially available compounds). The possible use of this ligand as a template was tested by applying the same validation set and protocol as for the HDAC6 pharmacophore (the ROC curve and AUC are reported in Fig. S1B). As for the pharmacophore approach, the use of N1*O descriptor for ranking generated the best AUC value, proving the efficacy of the constraint used. However, when the Glob-Prod descriptor was used, an AUC of about 0.5 was obtained. Although this result could be discouraging, it could be related to the nature of the available validation dataset (Table S2). Indeed, a deeper inspection of the screening results showed that in the LBVS, due to the large size and MW of the template, the small active molecules resulted too penalized when a global descriptor score (such as Glob-Prod) was used. Moreover, among the 84 collected compounds forming the validation dataset, none of them was active in the picomolar range. Thus, we decided to maintain the same approach reported for the PBVS. Therefore, the HBD constraint and Glob-Prod score rank were used. The FLAP ligand-based pre-filtering run allowed the identification of 1,373 compounds with a Glob-Sum higher than 0.5. These ligands were submitted to a second virtual screening round, taking into account GRID information calculated on SPECS compounds for a more accurate selection. The virtual screening results were ranked by Glob-Prod, and the 200 top-ranked compounds were selected for further inspection (Table S5).

### Selection of the compounds from PBVS and LBVS for *in vitro* testing

The top-ranked compounds from both PBVS and LBVS were carefully inspected. For selection, priority to compounds with ZBGs other than HA was given. First, 14 common compounds, identified by both approaches, were selected for *in vitro* testing. 26 compounds with structural diversity at the potential ZBG were also selected, making a total of 40 candidate compounds (Table S6). Their solubility was predicted using a PCA analysis using the VolSurf+ software^43^. The 40 molecules were projected in the solubility Volsurf+ model, and appeared to be in the same range as the 93 HDAC inhibitors characterizing the HDAC ChEMBL dataset (Fig. S2).

### HDAC isoform selectivity *in vitro*

**Figure 4:**
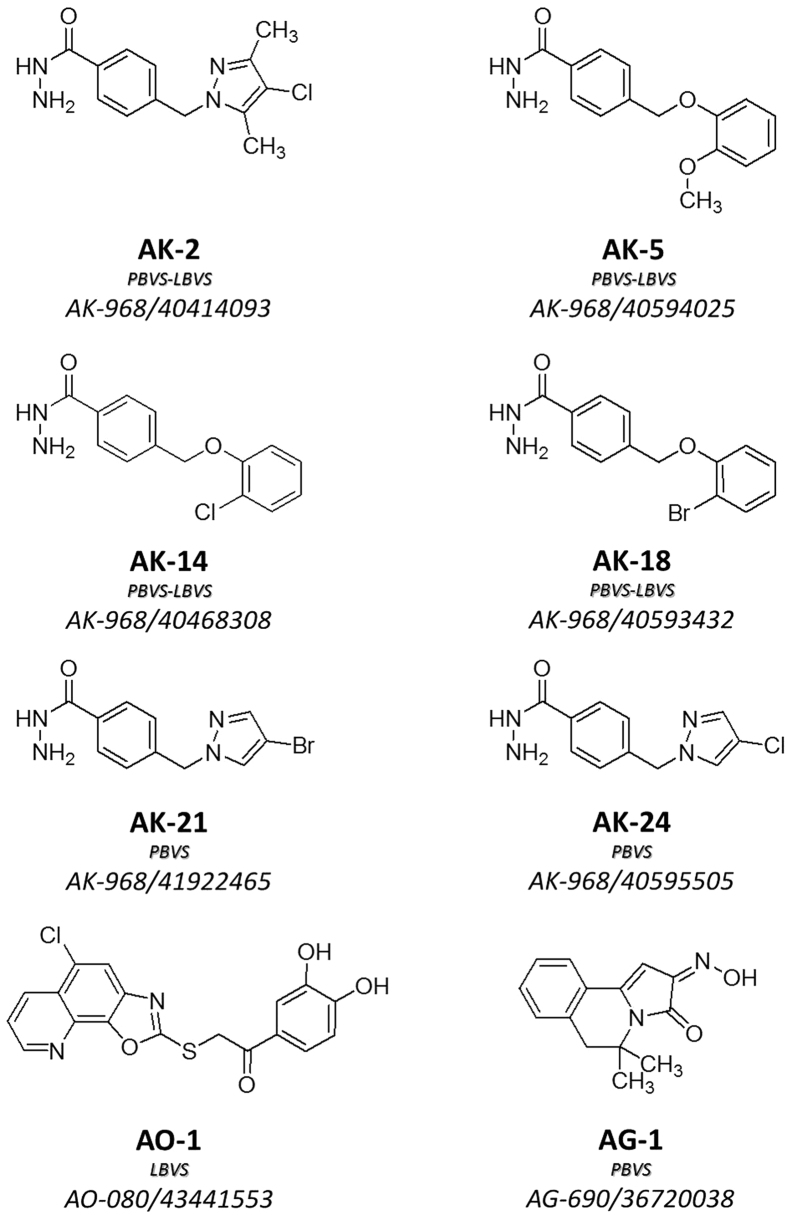
Chemical structure of the selected virtual screening hits from the SPECS database. The approach used for retrieving each compound is highlighted. PBVS as pharmacophore-based virtual screening; LBVS as ligand-based virtual screening.

The 40 compounds were screened on HDAC2, 8, 4 and 6 enzymes to assess their inhibitory activity. A two-step strategy was applied. First, an enzymatic screening campaign was performed for the 40 molecules on HDAC6. Compounds with inhibition higher than 50 percent when tested at 100 μM were retained for further inspection. HDAC screening using a pool of class I HDAC enzymes contained in HeLa nuclear extracts was then performed. A total of eight non HA-compounds having low IC_50_ values for HDAC6 inhibition (<100 μM) and low or no activity on HeLa nuclear extracts were identified. Their full selectivity profile was then determined on HDAC isoforms 2, 8, and 4 (Table 1). Among the eight selected compounds, three new ZBGs were identified (Fig. 4): six hydrazide-based compounds (AK series), one catechol derivative (AO-1), and one hydroxyimino molecule (AG-1). The best HDAC6-selective profile was achieved within the hydrazide series (Table 1). To the best of our knowledge, the capacity of hydrazides to inhibit HDACs has not been demonstrated so far^27^. On the contrary, hydrazides have been described as inactive towards HDACs^44^,^45^.

### Tubulin acetylation and cytotoxic properties of the most selective HDAC6 inhibitor AK-14

**Figure 5:**
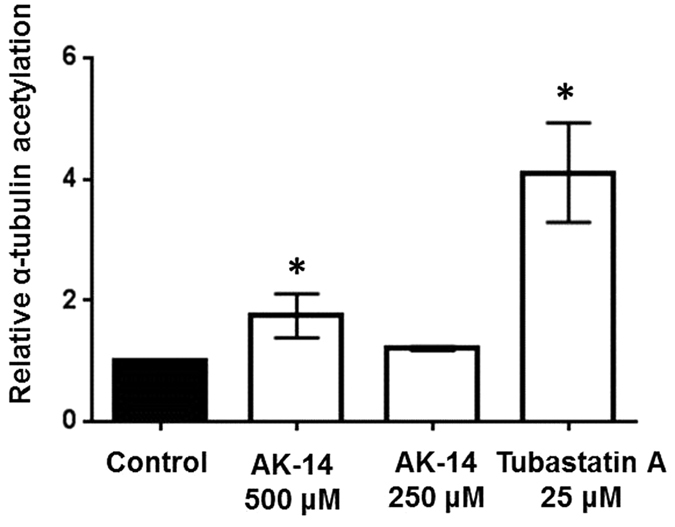
Relative α-tubulin acetylation in HeLa cells treated with AK-14 and Tubastatin A (*P < 0.05, two-sided t-test). Results are presented as the mean of 3 independent experiments in triplicate with error bars representing the standard deviation (SD).

**Figure 6:**
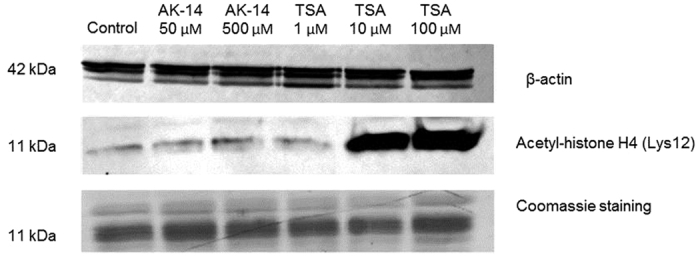
Western blot for Histone H4 acetylation levels in HeLa cells treated with AK-14 and Trichostatin A (TSA).

AK-14 was the most potent and selective compound obtained from the PBVS and LBVS campaign (Table 1). In order to determine if this compound is able to promote HDAC6 inhibition in cells, tubulin acetylation levels were measured in HeLa cells. AK-14 was able to significantly increase the levels of acetylated tubulin at 500 μM, with a less remarkable effect at 250 μM (Fig. 5). This can be explained by the potency of the HDAC6 inhibition by AK-14 (HDAC6 IC_50_ = 12.8 μM) compared to the selective HDAC6 inhibitor Tubastatin A (12.2 nM)^34^. Indeed, Tubastatin A is also able to induce tubulin acetylation at 25 μM, a concentration which represents ~2000-fold its HDAC6 IC_50_. In the case of AK-14, concentrations higher than 500 μM (~40-fold its HDAC6 IC_50_) cannot be tested due to solubility issues. More importantly, AK-14 did not change the acetylation profile of histone H4 in HeLa cells at 500 μM (Fig. 6), suggesting that the selective HDAC6 inhibition over class I HDACs is kept in living cells. The cytotoxic IC_50_ values were then determined for AK-14 towards HeLa and HEK 293 cells, using both Tubastatin A and Trichostatin A as positive controls (Table 2). In both cell lines, AK-14 showed low cytotoxicity. Usually, specific HDAC6 inhibitors (cf. Tubastatin A, Table 2) are less toxic than the pan-HDAC inhibitors (cf. Trichostatin A, Table 2). It is noteworthy that AK-14 was even less toxic than Tubastatin A, despite the fact that they are both selective HDAC6 inhibitors. However, this lower potency seems to be linked to the fact that AK-14 is also enzymatically less potent than Tubastatin A.

### Passive membrane permeation of the most HDAC6 selective inhibitor AK-14

HDAC6 selective inhibition has been found to be beneficial in neurodegenerative conditions^3^,^6^,^7^,^8^. This requires that HDAC6 inhibitors have the capability to cross the selective blood-brain barrier (BBB). The trans cellular passive BBB permeability potential of the low cytotoxic AK-14 was evaluated using PAMPA assays (PAMPA-BBB)^46^. The effective passive permeability value (P_e_) was calculated according the two-way flux equations obtained from the Fick’s law^47^,^48^. AK-14 demonstrated the capability to significantly cross the lipidic artificial membrane (P_e_ = 6.5 ± 0.9 cm/s), having a positive P_e_ value associated with the ability of such compounds to passively cross the BBB barrier (Table 1).

### Structural hypotheses of HDAC6 selective inhibition by AK-14

To explore the possible structural basis of AK-14 selective HDAC6 inhibition, a homology model of the HDAC6 catalytic domain^34^ was used for docking calculations. AK-14 was docked into the HDAC6 catalytic pocket using GOLD 5.2 (CCDC, Cambridge, UK) with a previously validated protocol^49^,^50^. The top-ranked docking pose (best structure of a 2 Å-cluster containing 13% docking solutions), was taken into account and submitted to 1,000 energy minimization cycles before analysis. According to our docking study, the hydrazide group of AK-14 interacts with the catalytic zinc ion in a bidentate way, while the carbonyl group makes a hydrogen bond with Tyr782 side chain. The phenyl ring characterizing the linker of AK-14 fits well the hydrophobic tunnel composed by bulky residues (Pro501, Pro608, and Phe680) through stable van der Waals interactions. The chloro-phenyl moiety results to be involved in π−π stacking and anion- π interactions with the aromatic and electronic rich residues surrounding the HDAC6 rim cavity (His500 and Asp567, respectively), and in hydrophobic interactions with Phe620 side chain. Interestingly, those residues were predicted to be involved in Tubastatin A stabilization into the HDAC6 binding site^34^. In 2010, Butler *et al.*^34^ hypothesized a wider rim channel for HDAC6, compared to other HDAC isoforms, as a main feature for selectivity (Table S7), in combination with the presence of a hydrophobic tunnel composed by bulky hydrophobic residues. The predicted AK-14 HDAC6 complex, reported in Fig. S3, seems to support those hypotheses.

## Discussion and Conclusions

HDAC6 is considered as a target with great therapeutic potential against aging-related diseases. In particular, selective HDAC6 catalytic inhibition has been associated with neuroprotection^3^,^6^,^7^,^8^. Indeed, missing crystallographic information on the HDAC6 catalytic site has limited the design of selective HDAC6 inhibitors. In this study, we decided to carry out an original computational study based on the structural and biological information of ligands described in the literature (Table S1). By analysing the ChEMBL collection in details, we actually realised that simultaneous inhibitory information related to four HDAC isoforms, representing class I (HDAC2, HDAC8) and class II HDACs (HDAC4, HDAC6), is reported only for 93 compounds, here referred to as the HDAC ChEMBL dataset (Table S2). We also noticed that 92 inhibitors were hydroxamate-bearing compounds. Only one compound (ChEMBL272980) was characterized by an anilide group, replacing the HA ZBG. This compound resulted to be more active on HDAC2 (IC_50_ = 0.29 μM) than on HDAC6 (IC_50_ = 10 μM). Based on the data reported in this dataset (Table S2), we decided to develop a pharmacophore model for HDAC6 catalytic inhibition to identify, in a second time, new selective HDAC6 inhibitors bearing non-HA ZBGs. The pharmacophore was generated by the alignment of three compounds, selected from the HDAC ChEMBL dataset, according to potency (IC_50_ < 100 nM), selectivity index (SI > 50), and MW (<600 Da). After model validation, a PBVS campaign of the SPECS database (203,891 compounds) was carried out. In parallel, a LBVS was conducted using the most potent HDAC6 inhibitor reported so far as a template (PMID_18642892_3, Fig. 2). 40 top ranked compounds bearing ZBG other than HA were selected and tested *in vitro*. The virtual screening campaign led to the discovery of eight novel non-hydroxamate inhibitors, all of them active in the low μM range towards HDAC6, while inactive on HDACs expressed in nuclear HeLa extracts (Table 1). Three new ZBGs were identified (Fig. 4): six hydrazide-based compounds (AK series), one catechol derivative (AO-1), and one hydroxyimino molecule (AG-1). The latter two compounds did not present important selectivity towards HDAC6 when tested on single HDAC isoforms 4 and 8. They did however present selectivity with respect to HDAC2 (SI > 50). Apart from specific interactions within active sites, this could be explained by the fact that HDAC2 displays the smallest binding pocket among the isoforms considered in this study (Table S7)^51^. This may prevent the accommodation of compounds possessing bulky moieties^12^,^34^. In this study, hydrazide-containing compounds were the most potent compounds against HDAC6 (Table 1). Literature data described hydrazides as inactive against HDACs^44^,^45^. Hydrazides share a few common properties with the HA group: they have similar pKa, and an identical number of hydrogen bond acceptors and donors. Hydrazides, however, are expected to be less susceptible to hydrolysis than HA because of their less electrophilic terminal amino group in comparison with the OH group in HA^52^. This feature confers an advantage to hydrazides in terms of pharmacokinetics. Several hydrazide molecules have been described as having favourable properties in terms of bioavailability^53^. Although they are good chelators, the presence of a hydrazide moiety is not sufficient to explain the observed selectivity profile of the selected hits towards HDAC6 (Fig. 4). It seems clear that the nature and the size of the linker are the main responsible for the observed selectivity. Indeed, HDAC6 seems to have the widest catalytic pocket among HDACs, according to homology modelling^34^. Nevertheless, it has been shown that plasticity in HDAC8 enables its catalytic pocket to undergo important conformational changes when in complex with ligands, mimicking HDAC6 features^12^. Whereas all of the linkers characterizing the AK series are characterized by the presence of a phenyl group, a methylene substituent linked to a five member heterocycle in *para* position (AK-2, AK-21, AK-24) impaired the selectivity (Table 1, Fig. 4). Interestingly, among the AK series, a relevant HDAC6 selectivity was maintained only when the phenyl group bears a methylene oxy-phenyl substitution in *para*. Replacement of the halogen in *ortho* on the phenyl ring (AK-14, AK-18) by a methoxy group (AK-5) led to a decrease of HDAC activity and selectivity (Table 1, Fig. 4). AK-14, the most potent and selective HDAC6 inhibitor, resulted to be top-ranked in both PBVS and LBVS approaches. This compound possesses a phenotypical behaviour consistent with its HDAC6 selectivity, as it was found to increase the levels of acetylated α-tubulin in HeLa cells (Fig. 5), with no effects on histone H4 acetylation (Fig. 6). Moreover, AK-14 showed low cytotoxicity towards HeLa and HEK 293 cells (Table 2), and good potential to passively cross the BBB. Taking all these considerations into account and considering the structural analysis pointing out possible elements of selectivity, AK-14 may be considered as a promising starting point for the design of potent hydrazide-based HDAC6 selective compounds for fighting against neurodegenerative conditions.

## Methods

### Dataset for pharmacophore generation

ChEMBL (https://www.ebi.ac.uk/chembl/) is an open database containing pharmacodynamic and pharmacokinetic information for bioactive compounds^54^. Compounds with available IC_50_ data obtained with enzymatic assays on HDAC2, HDAC8, HDAC4, and HDAC6 isoforms were selected for this study (accessed in January 2015). Molecules reviewed from Dallavalle *et al.*, having a selective HDAC6 inhibitory activity with respect to the other three isoforms herein considered, but not present in ChEMBL, were also taken into account^55^. HDAC inhibitors from the dataset were considered active toward a specific isoform when displaying an IC_50_ lower or equal to 10 μM, and inactive when displaying an IC_50_ higher or equal to 100 μM.

A database was then generated within the FLAP package (Molecular Discovery, UK, www.moldiscovery.com)^56^. Chemical structures were first imported in FLAP as SMILE strings. For each compound, 50 low energy conformers were generated, considering a protomeric state with greater than 20% abundance at pH 7.4. For each of these conformations, the molecular interaction fields (MIF) for H, O, N1, and DRY GRID probes were calculated at a 0.75 Å grid resolution, and FLAP fingerprints generated.

### Pharmacophore building

The FLAPpharm module of the FLAP package^39^ was used for pharmacophore generation. Three selected compounds were aligned in their most abundant protonation states according to MOKA predictions (Molecular Discovery, UK, www.moldiscovery.com)^57^. The top five conformations for each molecule, representing the most pharmacophorically similar conformers to the conformations found in the rest of the dataset, were retained for the generation of potential pharmacophore models (“fast” option). Each model consists of a molecular alignment from which common interacting features are expressed in terms of common pharmacophoric interaction fields (PIFs), common atom-centred pseudopharmacophoric interaction fields (pseudoPIFs), and common pharmacophoric points at the centroid of pseudoPIFs^39^. Pharmacophores were evaluated according to the S-score value. No constraints were applied in pharmacophore generation.

### Pharmacophore-based virtual screening (PBVS)

The SPECS database was downloaded from the website http://www.specs.net/. Compounds available in at least 10 mg amounts, accessed in January 2015, were filtered by molecular weight (MW ≤ 600 Da). The resulting 203,891 compounds were imported in the FLAP environment for dataset generation. The database included 25 possible conformers for each SPECS molecule, with a protomeric state over 20% abundancy at pH 7.4. As a first step, a FLAP pre-screening was performed in bit-string mode using the best pharmacophore model as a template. A hydrogen-bond donor (HBD) constraint was applied, as reported in Fig. 3. A similarity score was assigned based on the similarity of quadruplets built on atomic coordinates. At this stage, MIFs are not taken into account. The Glob-Sum similarity score (cut-off ≥ 0.25) was used for selecting 1,791 compounds from the SPECS database. This reduced dataset was again screened against the best pharmacophore model, this time taking into account information coming from the MIFs calculated for each SPECS compound. The accuracy of the FLAP algorithm for screening was set as “normal” and constraints were kept. The top ranked 200 compounds, according to the Glob-Prod similarity score, were selected for further inspection.

### Ligand-based virtual screening (LBVS)

The most potent HDAC6 inhibitor reported in the literature so far was used as a template for a LBVS in FLAP (Fig. 2)^55^. The SPECS dataset, previously used for the PBVS, was screened against the ligand. A pre-filtering approach, with the accuracy set as “fast” and with a constraint on the hydrogen-bond donor features, identified 1,373 SPECS compounds (Glob-Sum ≥ 0.25) that were then used to generate a new FLAP database, for a more accurate screening. This time, the accuracy of the FLAP algorithm screening was set as “normal” using MIF information. A HBD constraint on the hydroxamic acid group was applied. The top ranked 200 compounds, according to the Glob-Prod similarity score, were selected for further inspection.

### *In vitro* enzymatic assays for HDAC activity

*In vitro* deacetylase activity was detected using the fluorophore release from adequate Fluor de Lys acetylated substrates upon deacetylase enzymatic activity (reagents purchased from Enzo Life Sciences, enzymes purchased from Sigma Aldrich or produced by the Reaction Biology Corporation, Malvern, PA, using the Reaction Biology HDAC Spectrum platform, www.reactionbiology.com). HeLa nuclear extracts were used as source of class I HDACs. Trichostatin A (a pan-HDAC inhibitor) was used as positive control. Compounds were tested in dose-response (2% DMSO, in HDAC assay buffer). For each tested concentration, a corresponding blank was included to detect fluorescence artefacts. Assays were conducted in duplicate in 96-well plates at 37 °C for 30 (HeLa nuclear extracts) or 60 (HDAC2, 8, 6 and 4) min. Enzymatic reactions were stopped by adding HDAC developer I or II (Enzo Life Sciences) to each well, and the plate was incubated at room temperature. The resulting fluorescence intensity was measured at 360/460 nm or 485/530 nm, using a FLx800 microplate reader (Biotek). Untreated enzyme wells were included in each experiment as the 100% deacetylase activity control. IC_50_ values were determined through a dose-response assay and plotted on GraphPad Prism 6.0.

### Tubulin acetylation ELISA assay and acetyl histone H4 Western blotting

The acetyl-α-tubulin sandwich ELISA Kit (Pathscan^®^ #7204, Cell Signalling) was used to determine tubulin acetylation levels, and Western blotting was used to evaluate histone H4 acetylation, both methods applied to lysates of treated and non-treated HeLa cells (ATCC CCL-2). Cells were cultured in DMEM medium, 4.5 g/L glucose, L-glutamine (Invitrogen), supplemented with 10% heat-inactivated fetal bovine serum (FBS, Biowest), 100 units/mL penicillin, and 100 μg/mL streptomycin, and 1% non-essential amino acids (Invitrogen) at 37 °C with 5% CO_2_. Prior to ELISA experiments, cells were seeded into 6-well plates at 150,000 cells/well and transfected with the HDAC6-GFP construct (pEGFP.N1-HDAC6, Addgene)^58^, using the Lipofectamine 2000 reagent (Invitrogen). Cells were then treated with test compounds, standards or vehicle (0.5% DMSO in culture medium) for 48 h. After the incubation time, cells were washed with cold DPBS (Invitrogen) and lysates were obtained by recovering cells in cold lysis buffer provided in the ELISA kit, supplemented with a protease inhibitor tablet (SigmaFast, Sigma Aldrich). Briefly, cells were harvested in DPBS and centrifuged. The cell pellet was suspended in 100 μL lysis buffer, vortexed, and kept on melting ice for 30 min. Lysates were then obtained by recovering the supernatant after a centrifugation step of 10 min at 11,000 g, 4 °C. Protein concentration was determined by fluorimetry using the Qubit protein assay kit (Invitrogen). Cell lysates were tested at 1 mg/mL following the instructions provided by the ELISA kit manufacturer. For the Western blotting, cells were seeded into 100 mm Petri dishes and treated for 6 h. After the incubation time, cells were washed with cold DPBS (Invitrogen) supplemented with a protease inhibitor tablet (Sigma Fast, Sigma Aldrich), and lysates were obtained by recovering cells in 100 μL cold lysis buffer (10 mM Hepes pH 7.9, 1.5 mM MgCl_2_, 10 mM KCl, 0.5 mM DTT, 1.5 mM PMSF, 0.2M HCl) the same way as described above. Cell lysates (10 μg) were loaded, subjected to SDS-PAGE, transferred onto PVDF membranes at pH 10, and blotted with specific antibodies: acetyl-histone H4 (Lys12) rabbit mAb #13944 (Cell Signaling) and β-Actin mouse mAb #3700 (Cell Signaling). A control SDS-PAGE was stained with Coomassie blue for the detection of total histone bands.

### Cytotoxicity

HeLa and HEK 293 cells (ATCC CRL-1573) were cultured as described above and seeded into 96-well plates at 400 cells/well. Test and standard compounds were added to plate wells in triplicate (0.05% DMSO in culture medium) in a range of six concentrations. Following 72 h of incubation, 80 mL/well of an activated XTT solution was added to the cells (1 mg/mL). The absorbance was measured at 450 nm after 2 h incubation at 37 °C. The growth of the compound-treated cells was compared with the growth of vehicle-treated control cells. IC_50_ values were calculated with GraphPad Prism 6.0.

### Parallel Artificial Membrane Permeability Assays (PAMPA)

The PAMPA experiment was performed according to the PAMPA-BBB method described by Di *et al.*^46^. Test compounds were dissolved at 20 mM in DMSO and then diluted 200 times using a buffer solution at pH 7.4 (prepared according to the Geigy table, containing 54 mM Na_2_HPO_4_ and 13 mM KH_2_PO_4_). The resulting solutions, containing the compounds at 100 μM (0.5% DMSO), represented the donor solutions at the initial time. A total of 280 μL of the donor solution were added in each well of a Teflon^®^ 96-well microplate (Millipore MSSACCEPTOR) forming the donor compartments. Each well of a hydrophobic polyvinylidene fluoride (PVDF) 96-well microtitre filter plate (Multiscreen^®^ MAIPN4550, 70% porosity, pore diameter 0.45 μm, cross-sectional area 0.3 cm^2^) was impregnated with 4 μL of a polar brain lipids solution (20 mg/mL in dodecane, PBL obtained from Avanti Polar Lipids), placed under a fume hood and subjected to constant shaking at 75 rpm for approximately 10 min to homogeneously lay the artificial membrane. The PVDF filter plate impregnated with the artificial membranes was then carefully placed on the previously prepared Teflon plate. Finally, the filter plate was filled with 280 μl of the previously described buffer (i.e. “acceptor solutions”) in order to form the PAMPA “sandwich”, and left undisturbed for 18 hours at room temperature. After incubation, the sandwich was dissociated, and the donor and acceptor solutions were directly transferred into black 96-well plates (MaxiSorp, Milian SA) and sealed with heat sealing foil (Waters) prior to UHPLC–UV analysis. Each tested compound was analysed in triplicate. The effective passive permeability value (P_e_) was calculated according the two-way flux equations, obtained from the Fick’s law^47^. The calculated effective passive permeability values P_e_ were considered relevant only if RSD < 15% as quality control was fulfilled. Two reference standards were randomly placed in each 96-well plate layout. Cimetidine and imipramine were selected for their known effective passive permeability values^46^. The UHPLC measurements were performed using an Acquity UPLC system (Waters), which included a binary pumping system, an auto-sampler with an injection loop volume of 10 μl, a UV–VIS diode array programmable detector and a column manager over a pre-column heater. The systems were controlled using Empower Software v2.0, and UV detection was performed from 200 to 400 nm (6 nm resolution). The chromatograms were extracted at the appropriate UV wavelengths for each compound tested. The injection volume was performed in full loop mode (10 μl), the column temperature was set at 40 °C and the flow rate was fixed at 0.5 mL/min. The binary system used two mobile phases: water mQ with 0.1% formic acid (A) that had been filtered at 0.22 μm and HPLC grade acetonitrile with 0.1% formic acid (B). An Acquity UPLC^®^ BEH C18 (1.7 μm, 2.1 × 30 mm; Waters, Milford, MA, USA) was used as the stationary phase. A generic linear gradient was applied from 98% of A and 2% of B to 100% B over a period of 2 min, followed by an isocratic step of 100% of B during 0.20 min. The total analysis time was 3 min, including a re-equilibration step. Adequate UHPLC/UV data treatments were ensured by checking the UV signals of the tested compounds in the donor compartment at initial time. Only UV areas that had a signal/noise ratio greater than 100 were considered relevant for accurate P_e_ measurements while signals of low intensity were considered irrelevant.

### Molecular docking

The structures of AK-14 and HDAC6 were prepared for docking in Sybyl X1-3 (Tripos Inc., St. Louis, Mo). HDAC6 was structurally aligned on HDAC8-SAHA co-crystal (PDB 1T69). Molecular docking was then carried out using GOLD 5.2 (Genetic Optimization for Ligand Docking, CCDC Cambridge, UK) keeping the protein fixed, while the ligand fully flexible. The binding site for docking was defined within a radius of 6 Å around the co-crystallized ligand from HDAC8. 50 ligand poses were generated with Preset options, and ranked according to the Chem PLP scoring function. The best-ranked ligand and HDAC6 lateral chains were then submitted to 1,000 minimization cycles in Sybyl X 1.3, using the Tripos force field, Gasteiger-Huckel charges, and Powell algorithm. Images were generated with Sybyl X 1.3.

## Additional Information

**How to cite this article**: Goracci, L. *et al.* A Rational Approach for the Identification of Non-Hydroxamate HDAC6-Selective Inhibitors. *Sci. Rep.* **6**, 29086; doi: 10.1038/srep29086 (2016).

## Supplementary Material

Supplementary Information

## Figures and Tables

**Figure 1:**
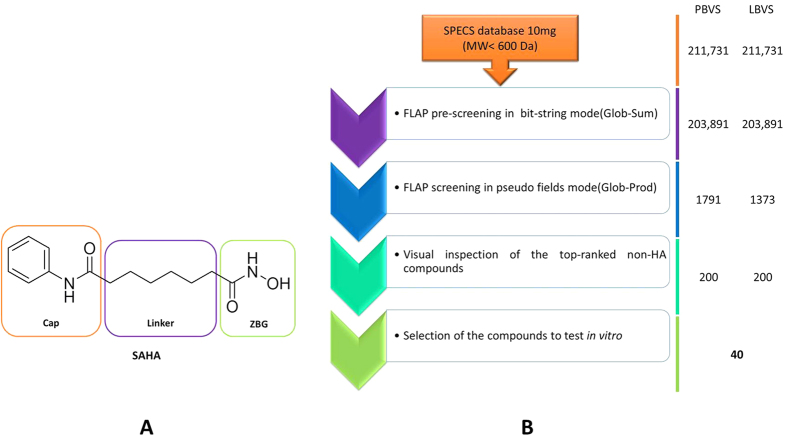
Prototypical pharmacophoric scheme for HDAC inhibition and the *in-silico* driven protocol adopted in this study. (**A**) Chemical structure of the FDA-approved HDAC inhibitor Vorinostat (SAHA): the prototypical pharmacophoric scheme for HDAC inhibition is highlighted. (**B**) Protocol for pharmacophore-based virtual screening (PBVS) and ligand-based virtual screening (LBVS) adopted in this study.

**Table 1:** Enzymatic activity profile of the 8 compounds selected from the virtual screening campaign.

ID *SPECS ID*	HeLa % of inhibition at 100 μM^a^	IC_50_ (μM)^b^			
		HDAC6	HDAC2	HDAC8	HDAC4
**AK-2** *AK-968/40414093*	10.0 ± 2.0	47.9 ± 4.7	>200	140.5 ± 11.5	>300
**AK-5** *AK-968/40594025*	5.8 ± 1.3	45.6 ± 1.5	>200	130.0 ± 9.0	>300
**AK-14**^c^ *AK-968/40468308*	2.7 ± 1.3	12.8 ± 5.4	>1000	>300	>300
**AK-18** *AK-968/40593432*	0.4 ± 3.9	16.4 ± 1.1	>1000	>300	>300
**AK-21** *AK-968/41922465*	14.7 ± 11.6	30.9 ± 2.1	201.7 ± 1.9	74.9 ± 0.8	>300
**AK-24** *AK-968/40595505*	33.3 ± 5.4	74.9 ± 5.2	>200	86.9 ± 0.7	>300
**AO-1** *AO-080/43441553*	20.7 ± 4.0	16.9 ± 4.9	>1000	35.1 ± 1.1	73.5 ± 6.0
**AG-1** *AG-690/36720038*	38.2 ± 1.1	21.7 ± 1.2	>1000	0.3 ± 0.005	110.5 ± 3.5
**Trichostatin A**	IC_50_ = 0.0106 ± 0.0027	0.0073 ± 0.0007	0.0097 ± 0.0009	0.41 ± 0.02	–
**TMP269**	–	–	–	–	0.40 ± 0.001

^a^Average enzyme percentage of inhibition of at least 3 independent experiments in duplicates.

^b^Average IC_50_ of at least 2 independent experiments in duplicates ± SEM.

^c^Permeability value obtained through PAMPA-BBB assay is P_e_ = 6.5 ± 0.9 cm/s (CNS +).

**Table 2:** Cytotoxic properties of AK-14.

	IC50 (μM)*	
	HeLa	HEK 293
**AK-14**	299.4 ± 56.1	34.5 ± 4.8
**Tubastatin A**	11.2 ± 0.4	2.4 ± 0.6
**Trichostatin A**	1.5 ± 0.3	0.3 ± 0.02

*Results are mean ± SD of 3 independent experiments in triplicate.
