# Supplementary material for: A Rational Approach for the Identification of Non-Hydroxamate HDAC6-Selective Inhibitors
Source: Sci Rep. 2016 Jul 12;6:29086. doi: 10.1038/srep29086 (PMC4941420; doi:10.1038/srep29086)
Supplement: Supplementary Information [file srep29086-s1.doc]

**Supplementary Information for:**

**A Rational Approach for the Identification Of Non-Hydroxamate HDAC6-Selective Inhibitors**

Laura Goracci^*a,b^, Nathalie Deschamps^*a^, Giuseppe Marco Randazzo^a^, Charlotte Petit^a^, Carolina Dos Santos Passos^a^, Pierre-Alain Carrupt^a^, Claudia Simões-Pires*^a^, Alessandra Nurisso*^ac^

^a^School of Pharmaceutical Sciences, University of Geneva, University of Lausanne

Quai Ernest‑Ansermet, 30, CH-1211, Geneva 4, Switzerland

^b^Laboratory for Cheminformatics and Molecular Modeling, Chemistry Department of Chemistry, Biology and Biotechnology University of Perugia, Via Elce di Sotto, 8, 06123 Perugia, Italy

^c^Département de Biochimie, Université de Montréal, H3C 3J7 Montréal, Québec, Canada

**Correspondence:**

Dr Alessandra Nurisso & Dr Claudia Simões-Pires

School of Pharmaceutical Sciences,

University of Geneva, University of Lausanne

CH-1211, Geneva 4 (Switzerland)

E-mail: alessandra.nurisso@unige.ch

claudia.avello@unige.ch

*Authors contributed equally to this work.

**Table S1.** Number (N) of HDAC inhibitors (HDAC2, 4, 6, 8) retrieved from the ChEMBL search.

|  | **HDAC2** | **HDAC4** | **HDAC6** | **HDAC8** |
| --- | --- | --- | --- | --- |
| **Compounds (N)** | 274 | 214 | 489 | 375 |
| **Active (≤10 µM)** | 192 | 134 | 406 | 288 |
| **Inactive (≥100 µM)** | 24 | 21 | 24 | 19 |
| **Compounds with data for all isoforms*** | 93 | | | |

**Table S2.** The HDAC ChEMBL dataset (93 compounds). The validation set (84 compounds) is also highlighted: active compounds are shaded in green; inactive compounds in red.

| **CMPD**  **ID** | **SMILES** | **HDAC2**  **IC50** | **HDAC4**  **IC50** | **HDAC6**  **IC50** | **HDAC8**  **IC50** | **Pubmed**  **ID** |
| --- | --- | --- | --- | --- | --- | --- |
| CHEMBL14227 | CCCC(=O)O | 12.000 µM | ≥ 100 µM | ≥ 100 µM | 15.000 µM | 21874153 |
| CHEMBL1469 | OC(=O)CCCc1ccccc1 | 65.000 µM | ≥ 100 µM | ≥ 100 µM | 93.000 µM | 21874153 |
| CHEMBL152162 | ONC(=O)CCc1ccccc1 | 2.720 µM | 33.330 µM | 0.448 µM | 2.350 µM | 23368884 |
| CHEMBL152665 | ONC(=O)Cc1ccccc1 | 33.330 µM | 33.330 µM | 1.100 µM | 11.620 µM | 23368884 |
| CHEMBL154574 | ONC(=O)\C=C\c1ccccc1 | 0.457 µM | 19.460 µM | 0.022 µM | 0.759 µM | 23368884 |
| CHEMBL16300 | ONC(=O)c1ccccc1 | 7.910 µM | 33.330 µM | 0.115 µM | 1.920 µM | 23368884 |
| CHEMBL1631910 | ONC(=O)CCCCCC1OCCCCCOc2ccccc2NC1=O | 1.930 µM | 2.580 µM | 0.016 µM | 0.643 µM | 21073160 |
| CHEMBL1631911 | ONC(=O)CCCCCC1OCCCCCCOc2ccccc2NC1=O | 1.570 µM | 1.200 µM | 0.010 µM | 1.100 µM | 21073160 |
| CHEMBL1631912 | ONC(=O)CCCCCC1OCCCCCCCOc2ccccc2NC1=O | 1.540 µM | 1.250 µM | 0.009 µM | 0.286 µM | 21073160 |
| CHEMBL1631913 | COc1cc2COc3ccccc3NC(=O)C(CCCCCC(=O)NO)OC\C=C/c(c2)c1 | 0.207 µM | 0.159 µM | 0.002 µM | 0.119 µM | 21073160 |
| CHEMBL1631914 | COc1cc2COc3ccccc3NC(=O)[C@H](CCCCCC(=O)NO)OC\C=C/c(c2)c1 | 0.158 µM | 0.079 µM | 0.001 µM | 0.198 µM | 21073160 |
| CHEMBL1631915 | COc1cc2COc3ccccc3NC(=O)[C@@H](CCCCCC(=O)NO)OC\C=C/c(c2)c1 | 0.382 µM | 0.284 µM | 0.010 µM | 0.206 µM | 21073160 |
| CHEMBL1631916 | COc1cc2CCCOC(CCCCCC(=O)NO)C(=O)Nc3ccccc3OCc(c2)c1 | 0.793 µM | 0.447 µM | 0.008 µM | 0.444 µM | 21073160 |
| CHEMBL1631917 | COc1cc2CCCO[C@@H](CCCCCC(=O)NO)C(=O)Nc3ccccc3OCc(c2)c1 | 0.268 µM | 0.118 µM | 0.003 µM | 0.136 µM | 21073160 |
| CHEMBL1631918 | COc1cc2CCCO[C@H](CCCCCC(=O)NO)C(=O)Nc3ccccc3OCc(c2)c1 | 0.425 µM | 0.454 µM | 0.004 µM | 0.091 µM | 21073160 |
| CHEMBL1672332 | CCCC(=O)NO | 9.000 µM | ≥ 100 µM | 2.000 µM | 26.000 µM | 21874153 |
| CHEMBL1767029 | CNC(=O)\C(=N/O)\CCCCCCC(=O)Nc1ccccc1 | ≥ 100 µM | 65.700 µM | ≥ 100 µM | 22.600 µM | 21417297 |
| CHEMBL1767030 | CNC(=O)\C(=N/O)\CCCCCCC(=O)Nc1ccc2ccccc2c1 | ≥ 100 µM | ≥ 100 µM | ≥ 100 µM | 65.000 µM | 21417297 |
| CHEMBL1767031 | CNC(=O)\C(=N/O)\CCCCCCC(=O)Nc1cnc2ccccc2c1 | ≥ 100 µM | ≥ 100 µM | ≥ 100 µM | ≥ 100 µM | 21417297 |
| CHEMBL1767032 | CNC(=O)\C(=N/O)\CCCCCCC(=O)NC1c2ccccc2c3ccccc13 | ≥ 100 µM | ≥ 100 µM | ≥ 100 µM | 58.400 µM | 21417297 |
| CHEMBL1767033 | CNC(=O)\C(=N/O)\CCCCCCC(=O)Nc1ccc(cc1)N(C)C | ≥ 100 µM | ≥ 100 µM | ≥ 100 µM | ≥ 100 µM | 21417297 |
| CHEMBL1767034 | CNC(=O)\C(=N/O)\CCCCCCC(=O)Nc1ccc(cc1)c2ccccc2 | ≥ 100 µM | ≥ 100 µM | ≥ 100 µM | ≥ 100 µM | 21417297 |
| CHEMBL1767035 | CNC(=O)\C(=N/O)\CCCCCCC(=O)Nc1cccc(Br)c1 | ≥ 100 µM | ≥ 100 µM | ≥ 100 µM | ≥ 100 µM | 21417297 |
| CHEMBL1767036 | CNC(=O)\C(=N/O)\CCCCCCC(=O)Nc1cccc(c1)c2ccccc2 | ≥ 100 µM | ≥ 100 µM | ≥ 100 µM | ≥ 100 µM | 21417297 |
| CHEMBL1767037 | CNC(=O)\C(=N/O)\CCCCCCC(=O)Nc1cccc(c1)c2ccc(cc2)C(=O)O | ≥ 100 µM | ≥ 100 µM | ≥ 100 µM | 29.400 µM | 21417297 |
| CHEMBL1767038 | CNC(=O)\C(=N/O)\CCCCCCC(=O)Nc1cccc(c1)c2cccnc2 | ≥ 100 µM | ≥ 100 µM | ≥ 100 µM | 76.700 µM | 21417297 |
| CHEMBL1767039 | CNC(=O)\C(=N/O)\CCCCCCC(=O)Nc1cccc(c1)c2ccc(Cl)nc2 | 69.700 µM | 58.900 µM | 93.500 µM | ≥ 100 µM | 21417297 |
| CHEMBL1767040 | CNC(=O)\C(=N/O)\CCCCCCC(=O)Nc1cccc(c1)c2cncc3cc(Cl)ccc23 | ≥ 100 µM | ≥ 100 µM | ≥ 100 µM | ≥ 100 µM | 21417297 |
| CHEMBL1767041 | CNC(=O)\C(=N\O)\CCCCCCNC(=O)c1cc(on1)c2cccc(NC(=O)OC(C)(C)C)c2 | ≥ 100 µM | ≥ 100 µM | ≥ 100 µM | ≥ 100 µM | 21417297 |
| CHEMBL1767042 | CNC(=O)\C(=N\O)\CCCCCCNC(=O)c1cc(on1)c2ccc(NC(=O)OC(C)(C)C)cc2 | ≥ 100 µM | ≥ 100 µM | ≥ 100 µM | ≥ 100 µM | 21417297 |
| CHEMBL1767043 | CNC(=O)\C(=N\O)\CCCCCCNC(=O)c1cc(on1)c2cccc(N)c2 | 33.000 µM | 38.900 µM | ≥ 100 µM | ≥ 100 µM | 21417297 |
| CHEMBL1767044 | CNC(=O)\C(=N\O)\CCCCCCNC(=O)c1cc(on1)c2ccc(N)cc2 | 48.100 µM | 48.800 µM | ≥ 100 µM | ≥ 100 µM | 21417297 |
| CHEMBL1767045 | CNC(=O)\C(=N/O)\CCCCC[C@H](N(Cc1ccc(OC)cc1)C(=O)OC(C)(C)C)C(=O)Nc2ccccc2 | ≥ 100 µM | ≥ 100 µM | 64.200 µM | ≥ 100 µM | 21417297 |
| CHEMBL1767046 | CNC(=O)\C(=N/O)\CCCCC[C@H](NCc1ccc(OC)cc1)C(=O)Nc2ccccc2 | 95.900 µM | 59.200 µM | 28.700 µM | 94.600 µM | 21417297 |
| CHEMBL1767047 | CNC(=O)\C(=N/O)\CCCCC[C@H](N(Cc1ccccc1)Cc2ccccc2)C(=O)Nc3ccccc3 | ≥ 100 µM | ≥ 100 µM | 35.800 µM | 87.000 µM | 21417297 |
| CHEMBL186311 | CCCC(CCC)C(=O)NO | ≥ 100 µM | ≥ 100 µM | 16.000 µM | 39.000 µM | 21874153 |
| CHEMBL1914702 | ONC(=O)CCCCCCNC(=O)c1cc(on1)c2ccc(NC(=O)c3cc(CN=[N+]=[N-])cc(c3)N=[N+]=[N-])cc2 | 0.780 µM | 0.432 µM | 0.047 µM | 0.651 µM | 21548582 |
| CHEMBL1914708 | ONC(=O)CCCCCCC(=O)Nc1cnn(Cc2cc(CN=[N+]=[N-])cc(c2)N=[N+]=[N-])c1 | 1.000 µM | 0.379 µM | 0.274 µM | 0.017 µM | 21548582 |
| CHEMBL216641 | COc1ccc2[nH]c(C)c(CC(=O)N[C@@H](CCCCCC(=O)NO)C(=O)NCCc3c([nH]c4ccccc34)c5ccccc5)c2c1 | 0.004 µM | 0.260 µM | 0.045 µM | 0.200 µM | 16987657 |
| CHEMBL2312164 | Cc1cccc(c1)C(=O)NOCCCCCC(=O)NO | 1.392 µM | 0.016 µM | 0.027 µM | 1.043 µM | 23252603 |
| CHEMBL2312167 | Cc1ccc(cc1C)C(=O)NOCCCCCC(=O)NO | 0.944 µM | 0.083 µM | 0.018 µM | 0.833 µM | 23252603 |
| CHEMBL2312168 | Cc1cc(C)cc(c1)C(=O)NOCCCCCC(=O)NO | 0.881 µM | 0.012 µM | 0.056 µM | 1.278 µM | 23252603 |
| CHEMBL2333338 | CN1CCN(CCNC(=O)c2ccc(cc2)C(=O)NO)CC1 | 16.790 µM | 33.330 µM | 1.360 µM | 20.910 µM | 23368884 |
| CHEMBL2333339 | ONC(=O)c1ccc(cc1)C(=O)NCCc2ccncc2 | 0.341 µM | 33.330 µM | 0.008 µM | 1.330 µM | 23368884 |
| CHEMBL2333340 | ONC(=O)c1ccc(cc1)C(=O)NCCc2ccccc2 | 0.607 µM | 33.330 µM | 0.004 µM | 1.150 µM | 23368884 |
| CHEMBL2333341 | ONC(=O)c1ccc(cc1)C(=O)NCc2ccccc2 | 0.381 µM | 33.330 µM | 0.007 µM | 1.030 µM | 23368884 |
| CHEMBL2333342 | ONC(=O)c1ccc(cc1)C(=O)Nc2ccccc2 | 0.321 µM | 33.330 µM | 0.037 µM | 0.689 µM | 23368884 |
| CHEMBL2333343 | CNC(=O)c1ccc(cc1)C(=O)NO | 1.610 µM | 33.330 µM | 0.028 µM | 1.950 µM | 23368884 |
| CHEMBL2333344 | ONC(=O)C1=CCCCC1 | 1.050 µM | 9.130 µM | 0.012 µM | 0.431 µM | 23368884 |
| CHEMBL2333345 | ONC(=O)C1CCCCC1 | 33.330 µM | 33.330 µM | 0.376 µM | 3.720 µM | 23368884 |
| CHEMBL2333346 | ONC(=O)C1=CCCC1 | 1.790 µM | 21.800 µM | 0.030 µM | 1.090 µM | 23368884 |
| CHEMBL2364628 | ONC(=O)CCCCCCNC(=O)c1cnc(nc1)N(c2ccccc2)c3ccccc3 | 0.048 µM | 7.000 µM | 0.005 µM | 0.100 µM | 23627282 |
| CHEMBL2381517 | ONC(=O)c1cccc(c1)C(=O)NCCc2ccccc2 | 9.000 µM | 33.000 µM | 0.036 µM | 0.120 µM | 23672185 |
| CHEMBL2381518 | ONC(=O)c1cccc(c1)C(=O)NCCc2cccnc2 | 20.000 µM | 33.000 µM | 0.059 µM | 0.420 µM | 23672185 |
| CHEMBL2381519 | ONC(=O)c1cccc(c1)C(=O)NCc2ccccc2 | 11.000 µM | 33.000 µM | 0.034 µM | 0.210 µM | 23672185 |
| CHEMBL2381520 | ONC(=O)c1cccc(c1)C(=O)Nc2ccccc2 | 30.000 µM | 33.000 µM | 0.057 µM | 0.110 µM | 23672185 |
| CHEMBL2381521 | CNC(=O)c1cccc(c1)C(=O)NO | 33.000 µM | 33.000 µM | 2.500 µM | 14.000 µM | 23672185 |
| CHEMBL2381522 | Cc1cccc(c1)C(=O)NO | 33.000 µM | 33.000 µM | 0.650 µM | 3.300 µM | 23672185 |
| CHEMBL2381523 | ONC(=O)c1cccc(CNc2ccccc2)c1 | 33.000 µM | 33.000 µM | 1.300 µM | 1.700 µM | 23672185 |
| CHEMBL2381524 | ONC(=O)c1cccc(\C=C\C(=O)Nc2ccccc2)c1 | 4.800 µM | 14.000 µM | 0.021 µM | 0.037 µM | 23672185 |
| CHEMBL2414098 | ONC(=O)CCCCCCc1nc2ccc(Br)cc2[nH]1 | 0.034 µM | 0.279 µM | 0.002 µM | 0.282 µM | 23906422 |
| CHEMBL2431862 | Cn1cccc1C(=O)NCc2ccc(cc2)C(=O)NO | 16.000 µM | 15.000 µM | 0.460 µM | 0.690 µM | 23964961 |
| CHEMBL2431901 | Cn1cccc1C(=O)N2CCc3cc(ccc3C2)C(=O)NO | 50.000 µM | 50.000 µM | 0.036 µM | 2.100 µM | 23964961 |
| CHEMBL2431902 | CC(C)(C)C(=O)N1CCc2cc(ccc2C1)C(=O)NO | 50.000 µM | 50.000 µM | 0.430 µM | 2.100 µM | 23964961 |
| CHEMBL2431906 | Cn1cccc1C(=O)N2Cc3ccc(cc3C2)C(=O)NO | 50.000 µM | 50.000 µM | 0.040 µM | 1.300 µM | 23964961 |
| CHEMBL2431907 | CC(C)(C)C(=O)N1Cc2ccc(cc2C1)C(=O)NO | 50.000 µM | 50.000 µM | 0.560 µM | 1.300 µM | 23964961 |
| CHEMBL2431912 | Cn1cccc1C(=O)N2CCCc3cc(ccc3C2)C(=O)NO | 50.000 µM | 50.000 µM | 1.480 µM | 0.890 µM | 23964961 |
| CHEMBL272980 | Nc1ccccc1NC(=O)c2ccc(CNc3nccc(n3)c4cccnc4)cc2 | 0.290 µM | 10.000 µM | 10.000 µM | 10.000 µM | 18570366 |
| CHEMBL356769 | OCc1ccc(cc1)[C@@H]2C[C@H](CSc3oc(c4ccccc4)c(n3)c5ccccc5)O[C@@H](O2)c6ccc(NC(=O)CCCCCCC(=O)NO)cc6 | 6.270 µM | 17.300 µM | 0.004 µM | 1.270 µM | 23627282 |
| CHEMBL470843 | Cl.ONC(=O)\C=C\c1ccc(cc1)c2cc(CN3CCOCC3)on2 | 13.200 µM | 11.900 µM | 0.175 µM | 0.127 µM | 19285395 |
| CHEMBL471041 | ONC(=O)\C=C\c1ccc(\C=N\OCc2c(F)c(F)c(F)c(F)c2F)cc1 | 11.000 µM | 38.800 µM | 0.661 µM | 1.290 µM | 19285395 |
| CHEMBL471042 | ONC(=O)\C=C\c1ccc(\C=N\OCc2ccc(cc2)[N+](=O)[O-])cc1 | 2.670 µM | 2.040 µM | 0.124 µM | 0.070 µM | 19285395 |
| CHEMBL471043 | COC(=O)c1ccc(CO\N=C\c2ccc(\C=C\C(=O)NO)cc2)cc1 | 15.800 µM | 12.900 µM | 0.094 µM | 0.603 µM | 19285395 |
| CHEMBL472631 | ONC(=O)\C=C\c1ccc(\C=N\OCCN2CCOCC2)cc1 | 27.700 µM | 16.100 µM | 0.303 µM | 0.235 µM | 19285395 |
| CHEMBL483254 | Cc1[nH]c2ccccc2c1CCNCc3ccc(\C=C\C(=O)NO)cc3 | 0.300 µM | 0.300 µM | 0.300 µM | 0.300 µM | 23672185 |
| CHEMBL484073 | ONC(=O)CCCCCNC(=O)\C=C\c1ccc2cccnc2c1 | 0.003 µM | 0.005 µM | 0.003 µM | 2.028 µM | 18672316 |
| CHEMBL484489 | ONC(=O)CCCCCNC(=O)\C=C\c1ccc(Cl)cc1F | 0.003 µM | 0.004 µM | 0.003 µM | 1.620 µM | 18672316 |
| CHEMBL485514 | ONC(=O)CCCCCNC(=O)C=C1c2ccccc2c3ccccc13 | 0.001 µM | 0.001 µM | 0.002 µM | 0.779 µM | 18672316 |
| CHEMBL490018 | C\C(=C/C(=O)NCCCCCC(=O)NO)\C=C\c1ccccc1 | 0.003 µM | 0.004 µM | 0.004 µM | 2.116 µM | 18672316 |
| CHEMBL55895 | ONC(=O)CCCc1ccccc1 | 0.600 µM | ≥ 100 µM | 0.500 µM | 4.000 µM | 21874153 |
| CHEMBL561483 | COc1cccc2c(c[nH]c12)C(CCCCCC(=O)NO)c3c[nH]c4c(OC)cccc34 | 0.440 µM | 0.220 µM | 0.003 µM | 0.180 µM | 19359173 |
| CHEMBL561604 | ONC(=O)CCCCCC(c1c[nH]c2ccc(cc12)[N+](=O)[O-])c3c[nH]c4ccc(cc34)[N+](=O)[O-] | 0.590 µM | 0.220 µM | 0.020 µM | 0.600 µM | 19359173 |
| CHEMBL564382 | ONC(=O)\C=C\c1ccc(cc1)C(c2c[nH]c3ccccc23)c4c[nH]c5ccccc45 | 3.900 µM | 4.800 µM | 4.900 µM | 0.730 µM | 19359173 |
| CHEMBL564876 | ONC(=O)CCCCCC(c1c[nH]c2ccccc12)c3c[nH]c4ccccc34 | 2.000 µM | 1.300 µM | 0.006 µM | 0.240 µM | 19359173 |
| CHEMBL594544 | OCC(\C=C\c1ccc(F)cc1)N2CCN(CC2)c3ncc(cn3)C(=O)NO | 0.018 µM | 0.207 µM | 0.224 µM | 0.030 µM | 19906529 |
| CHEMBL598797 | COc1cc2ncnc(Nc3cccc(c3)C#C)c2cc1OCCCCCCC(=O)NO | 0.013 µM | 0.013 µM | 0.005 µM | 0.080 µM | 20143778 |
| CHEMBL609583 | ONC(=O)c1cnc(nc1)N2CCN(CC2)S(=O)(=O)c3ccc4ccccc4c3 | 0.009 µM | 0.005 µM | 0.066 µM | 0.027 µM | 19906529 |
| Aurothioglucose | SC1OC(CO)C(O)C(O)C1O | ≥ 100 µM | ≥ 100 µM | ≥ 100 µM | ≥ 100 µM | 22047054 |
| Ebselen | [Se]1N(C(=O)c2c1cccc2)c1ccccc1 | ≥ 100 µM | ≥ 100 µM | ≥ 100 µM | ≥ 100 µM | 22047054 |
| GNTI | O1c2c3C45C1c1[nH]c6c(cc(\[NH+]=C(\N)/N)cc6)c1CC4(O)C(N(CC5)CC1CC1)Cc3ccc2O | ≥ 100 µM | ≥ 100 µM | ≥ 100 µM | ≥ 100 µM | 22047054 |
| NSC-95397 | S(CCO)C1=C(SCCO)C(=O)c2c(cccc2)C1=O | ≥ 100 µM | ≥ 100 µM | ≥ 100 µM | ≥ 100 µM | 22047054 |
| U-73122 | O(C)c1cc2CCC3C4CCC([NH2+]CCCCCCN5C(=O)C=CC5=O)C4(CCC3c2cc1)C | ≥ 100 µM | ≥ 100 µM | ≥ 100 µM | ≥ 100 µM | 22047054 |
| ACY-1215 | O=C(NCCCCCCC(=O)NO)c1cnc(nc1)N(c1ccccc1)c1ccccc1 | 48 µM | ≥ 100 µM | 4.6999998 | ≥ 100 µM | 22262760 |

**Table S3.** Isoform selectivity (ratio) for the compounds selected for HDAC6 pharmacophore building.

| **CMPD**  **ID** | **HDAC2/HDAC6** | **HDAC4/HDAC6** | **HDAC8/HDAC6** |
| --- | --- | --- | --- |
| CHEMBL2333340 | 151.75 | 8332.5 | 287.5 |
| CHEMBL2431901 | 1388.9 | 1388.9 | 58.33 |
| CHEMBL2431906 | 169.2 | 84.6 | 69.2 |

**Table S4.** List of the 200 best-ranked compounds obtained with the pharmacophore based virtual screening approach. Highest Glob-Prod value is 0.492 whereas the lowest is 0.348.

| **SPECs ID** | **SPECs ID** | **SPECs ID** | **SPECs ID** | **SPECs ID** |
| --- | --- | --- | --- | --- |
| AF_886_30580032 | AK_968_41922469 | AP_263_43418623 | AP_263_41011952 | AK_968_41922430 |
| AE_413_30123024 | AK_968_41922478 | AP_263_43418626 | AP_263_41012033 | AK_968_41922431 |
| AP_263_11633946 | AK_968_41922481 | AP_263_43418629 | AG_690_37012079 | AK_968_41922432 |
| AG_690_11633949 | AK_968_41922482 | AP_263_43418631 | AN_465_43369360 | AK_968_41922434 |
| AG_690_11633959 | AK_968_41922494 | AK_968_40606028 | AI_204_31709007 | AK_968_41922445 |
| AG_690_11633970 | AK_968_41922496 | AP_263_43418642 | AN_465_43369729 | AK_968_41922448 |
| AG_205_12900229 | AK_968_41922500 | AP_263_43418644 | AN_465_43369879 | AK_968_41922459 |
| AA_516_12432222 | AK_968_41922508 | AP_263_43418649 | AP_263_43371122 | AK_968_41922464 |
| AA_516_12432234 | AK_968_41922510 | AP_263_43418653 | AF_962_31930030 | AK_968_41922465 |
| AA_516_12432244 | AK_968_41922513 | AK_968_41925112 | AP_263_43371126 | AK_968_40605942 |
| AL_182_12959014 | AK_968_41922524 | AK_968_41925139 | AK_968_40707827 | AK_968_41924492 |
| AG_690_11350509 | AO_359_40796545 | AK_968_41925718 | AK_968_40708249 | AK_968_40605545 |
| AG_803_12478010 | AK_968_40414093 | AK_968_41925719 | AG_690_37071103 | AG_205_36285041 |
| AF_886_30565022 | AK_968_40449222 | AK_968_41925721 | AP_970_41681515 | AP_263_43418479 |
| AF_886_30565063 | AK_968_40468126 | AK_968_41925722 | AP_970_41681517 | AP_263_43418481 |
| AG_690_36720038 | AK_968_40468162 | AK_968_41925723 | AN_465_41681651 | AP_263_43418482 |
| AJ_292_12675477 | AK_968_40468187 | AK_968_41925725 | AP_970_43374184 | AK_968_40605918 |
| AG_690_11020052 | AK_968_40468195 | AK_968_41925726 | AO_476_42871107 | AK_968_40605919 |
| AO_854_03842028 | AK_968_40468222 | AK_968_41925727 | AO_476_42871108 | AK_968_40605927 |
| AH_034_08839011 | AK_968_40468308 | AK_968_41925728 | AO_476_42871109 | AK_968_40605928 |
| AH_262_34399036 | AK_968_40468322 | AL_281_15562031 | AN_465_43461076 | AK_968_40605929 |
| AG_690_11764110 | AK_968_40468352 | AL_281_42504224 | AG_690_32531006 | AO_476_43362743 |
| AG_205_13630017 | AJ_333_36117045 | AT_051_43421444 | AO_548_43379595 | AO_476_43362744 |
| AN_329_11450268 | AJ_333_36117058 | AK_968_40641674 | AP_906_41027216 | AE_848_30897008 |
| AG_690_12883799 | AK_968_40514441 | AN_465_42538361 | AI_555_32919016 | AN_465_42766923 |
| AQ_358_40177668 | AN_648_14239001 | AN_465_42538920 | AI_555_32919020 | AN_465_42767066 |
| AK_968_40184255 | AP_263_43417823 | AG_205_36566055 | AO_854_43464398 | AI_204_31681037 |
| AQ_911_34094041 | AK_968_40581053 | AK_968_40642424 | AN_329_41044392 | AN_465_43369096 |
| AK_968_40211882 | AK_968_40593178 | AT_051_43422466 | AK_693_43467006 | AG_205_37007288 |
| AN_465_43411311 | AK_968_40593187 | AF_399_41438093 | AG_205_37223058 | AN_465_43369129 |
| AP_906_41880914 | AK_968_40593432 | AF_615_41584041 | AP_970_42167283 | AN_465_43369159 |
| AK_968_40355672 | AK_968_40594025 | AA_516_30012006 | AK_968_15359047 | AP_263_41011944 |
| AK_968_40355793 | AK_968_40594215 | AE_534_30057008 | AF_399_37285065 | AP_518_41793425 |
| AK_968_41922011 | AK_968_40594263 | AE_473_30079010 | AK_968_15359423 | AS_871_43476671 |
| AK_968_41922014 | AK_968_40595078 | AF_399_43344877 | AF_399_37411020 | AS_871_43476813 |
| AK_968_41922405 | AK_968_40595175 | AH_487_40686655 | AN_465_43013625 | AO_365_43401490 |
| AK_968_41922413 | AK_968_40595318 | AO_476_43362740 | AS_871_43475557 | AG_690_15427100 |
| AK_968_41922415 | AK_968_40595505 | AO_476_43362741 | AA_516_33241014 | AS_871_43477395 |
| AK_968_41922423 | AK_968_40605219 | AO_476_43362742 | AI_204_33263011 | AH_262_33733002 |
| AK_968_41922428 | AH_262_33733014 | AG_690_33774024 | AG_670_33930027 | AP_263_41011946 |

**Table S5.** List of the 200 best-ranked compounds obtained with the ligand based virtual screening approach. Highest Glob-Prod value is 0.428 whereas the lowest is 0.300.

| **SPECs ID** | **SPECs ID** | **SPECs ID** | **SPECs ID** | **SPECs ID** |
| --- | --- | --- | --- | --- |
| AG_205_11868084 | AK_968_41922494 | AS_871_40612138 | AO_022_43451970 | AS_871_42110661 |
| AE_641_00605015 | AK_968_41922499 | AG_690_36333059 | AM_900_15050043 | AH_487_13581012 |
| AM_879_12129057 | AK_968_41922500 | AK_968_41925112 | AM_900_15050058 | AQ_390_42131119 |
| AE_641_01634026 | AK_968_41922510 | AO_081_15571320 | AN_655_40688220 | AG_205_37136074 |
| AH_034_32858003 | AP_263_43241304 | AN_989_40872717 | AO_022_43452423 | AK_918_37182006 |
| AG_690_10516036 | AF_399_40804836 | AK_968_41925718 | AO_022_43452461 | AK_918_37182007 |
| AN_979_13514021 | AK_968_40414093 | AK_968_41925719 | AI_204_31677020 | AQ_750_41790303 |
| AG_690_11767698 | AE_641_15486017 | AK_968_41925721 | AO_022_43452495 | AS_871_43476226 |
| AE_848_11827085 | AK_968_40449222 | AK_968_41925722 | AO_081_40694601 | AN_465_41078984 |
| AG_690_12374014 | AK_968_40468126 | AK_968_41925725 | AG_690_40696439 | AP_853_43386911 |
| AG_690_09689054 | AK_968_40468187 | AK_968_41925727 | AG_690_40696447 | AG_690_40749875 |
| AG_690_09696022 | AK_968_40468195 | AN_465_43421602 | AO_022_43452972 | AG_690_40749876 |
| AO_082_13829010 | AK_968_40468222 | AN_465_43421603 | AO_022_43453191 | AG_690_40750079 |
| AK_968_40184255 | AK_968_40468308 | AF_399_15599257 | AO_022_43453199 | AG_690_40750080 |
| AF_399_40769025 | AG_690_36103015 | AK_968_40642638 | AO_022_43453212 | AF_399_37305012 |
| AG_690_15432850 | AF_399_14201003 | AK_968_40642702 | AF_399_15128304 | AF_399_37305013 |
| AN_988_41873680 | AO_476_43417544 | AO_080_43441553 | AQ_088_42013898 | AO_009_37312002 |
| AK_918_34356050 | AQ_750_42422256 | AJ_292_14694006 | AG_690_40700189 | AO_009_37312004 |
| AO_548_43196489 | AK_968_40514441 | AG_690_36682021 | AN_465_43369489 | AN_988_37365023 |
| AN_329_40256075 | AK_968_40593178 | AM_900_14781022 | AO_022_43453921 | AN_988_37365029 |
| AN_465_43411356 | AK_968_40593187 | AN_988_40679737 | AO_022_43454097 | AN_988_37365038 |
| AN_465_43411357 | AK_968_40593432 | AN_988_40679740 | AO_022_43454444 | AN_988_37365043 |
| AE_848_34511056 | AK_968_40594025 | AN_988_40679742 | AO_022_43454583 | AN_988_37365058 |
| AN_698_40780662 | AK_968_40594215 | AN_988_40679746 | AO_022_43454595 | AP_064_42208105 |
| AN_698_40780791 | AK_968_40594263 | AN_648_14914328 | AP_970_41681554 | AP_064_42208253 |
| AN_698_40781644 | AK_968_40595175 | AN_023_41957972 | AO_476_15188007 | AQ_768_41832102 |
| AN_698_40781705 | AK_968_40595318 | AO_081_41965592 | AO_022_43454715 | AG_690_40751381 |
| AN_698_40782114 | AK_968_40595682 | AE_641_30115058 | AO_022_43454927 | AG_690_40751404 |
| AK_968_40336998 | AK_968_40595687 | AN_698_41982065 | AE_848_37031130 | AS_871_43478036 |
| AN_988_40788226 | AP_853_43260811 | AN_698_41984866 | AN_648_15240063 | AS_871_43478043 |
| AG_205_34707042 | AN_465_40839063 | AN_698_41985442 | AO_022_43455312 | AQ_088_41136935 |
| AK_968_41922405 | AP_853_43261616 | AE_641_30156001 | AO_022_43455490 | AQ_088_41137421 |
| AK_968_41922413 | AK_968_40605219 | AE_641_30156002 | AO_022_43455513 | AQ_088_41137436 |
| AK_968_41922428 | AF_399_15539034 | AQ_088_42695095 | AP_970_43374095 | AQ_088_41137447 |
| AK_968_41922430 | AK_968_40605671 | AF_399_15034092 | AN_989_41695732 | AK_918_40033071 |
| AK_968_41922431 | AF_399_40850614 | AN_465_41995495 | AN_465_43460973 | AG_690_40754410 |
| AK_968_41922448 | AK_968_40605919 | AN_465_41995500 | AN_465_43461001 | AG_670_33930027 |
| AK_968_41922464 | AK_968_40605927 | AP_906_42715910 | AG_690_37071065 | AG_690_40755577 |
| AK_968_41922469 | AK_968_40605929 | AE_848_30897008 | AK_968_41024608 | AG_690_40756330 |
| AK_968_41922481 | AF_399_40852818 | AK_968_40610213 | AG_690_40135330 | AO_476_43407143 |

**Table S6. 40 compounds from the pharmacophore based and ligand-based virtual screening selected for *in vitro* testing.**

| **SPECS**  **ID** | **STRUCTURE** | **METHOD** | **HDAC6 % of inhibition at 100 µM*** | **HeLa % of inhibition at 100 µM*** | **IC_50_**  **HDAC6*** |
| --- | --- | --- | --- | --- | --- |
| **AK-1**  *AK-968/40468222* |  | PBVS  LBVS | 18.3 ± 0.1% | ND* | ND |
| **AK-2**  *AK-968/40414093* |  | PBVS  LBVS | **58.0 ± 1.5%** | 10.0  ± 3.5% | **47.9**  **± 8.1 µM** |
| **AK-3**  *AK-968/41925726* |  | PBVS | 0.1 ± 2.3% | ND | ND |
| **AK-4**  *AK-968/41922459* |  | PBVS | 3.0 ± 0.3% | ND | ND |
| **AK-5**  *AK-968/40594025* |  | PBVS  LBVS | **64.4 ± 1.3 %** | 5.8 ± 2.2% | **45.6**  **± 2.2 µM** |
| **AK-6**  *AK-968/40605219* |  | PBVS  LBVS | 15.3 ± 0.5% | ND | ND |
| **AK-7**  *AK-968/40605918* |  | PBVS | 31.1 ± 0.5%  Self-fluorescence | ND | ND |

| **AK-8**  *AK-968/40514441* |  | LBVS | 32.5 ± 8.8%  Solubility issues  Self-fluorescence | ND* | ND |
| --- | --- | --- | --- | --- | --- |
| **AK-9**  *AK-968/40641674* |  | PBVS | 8.0 ± 4.6% | ND | ND |
| **AK-10**  *AK-968/40355793* |  | PBVS | 13.9 ± 17.9% | ND | ND |
| **AK-11**  *AK-968/41922464* |  | PBVS  LBVS | 5.3 ± 19.9% | ND | ND |
| **AK-12**  *AK-968/40449222* |  | PBVS  LBVS | 28.2 ± 16.7% | ND | ND |
| **AK-13**  *AK-968/41922499* |  | LBVS | 23.7 ± 9.3% | ND | ND |
| **AK-14**  *AK-968/40468308* |  | PBVS  LBVS | **70.7 ± 6. %** | 2.7 ± 2.2% | **12.8**  **± 9.3 µM** |
| **AK-15**  *AK-968/40468195* |  | PBVS  LBVS | 42.1 ± 23.0% | -2.6 ± 3.0% | ND* |

| **AK-16**  *AK-968/40468187* |  | PBVS  LBVS | 36.9 ± 11.3% | ND | ND |
| --- | --- | --- | --- | --- | --- |
| **AK-17**  *AK-968/40594215* |  | PBVS  LBVS | 35.5 ± 10.5% | ND | ND |
| **AK-18**  *AK-968/40593432* |  | PBVS  LBVS | **69.0 ± 2.4%** | 0.4 ± 6.8% | **16.4**  **± 2.2 µM** |
| **AK-19**  *AK-968/41922478* |  | PBVS | 18.1 ± 4.0% | ND | ND |
| **AK-20**  *AK-968/41922496* |  | PBVS | 22.3 ± 8.7% | ND | ND |
| **AK-21**  *AK-968/41922465* |  | PBVS | **67.9 ± 7.8%** | 14.7 ± 20.1% | **30.9**  **± 3.6 µM** |
| **AK-22**  *AK-968/40593187* |  | PBVS  LBVS | 25.4 ± 9.7% | ND | ND |
| **AK-23**  *AK-968/40605942* |  | PBVS | 20.2 ± 10.9% | ND* | ND |
| **AK-24**  *AK-968/40595505* |  | PBVS | **63.1**  **± 12.2%** | 33.3 ± 9.4% | **74.9**  **± 9.0 µM** |
| **AK-25**  *AK-968/40595318* |  | PBVS  LBVS | 29.1  ± 10.3 % | ND | ND |
| **AO-1**  *AO-080/43441553* |  | LBVS | **66.1 ± 6.9%** | 20.7 ± 6.9% | **16.9**  **± 8.5 µM** |
| **AO-2**  *AO-476/43407143* |  | LBVS | -3.3 ± 5.4% | ND | ND |
| **AQ-1**  *AQ-750/41790303* |  | LBVS | -16.4 ± 9.3%  Solubility issues  Self-fluorescence | ND | ND |
| **AN-1**  *AN-989/40872717* |  | LBVS | -43.0  ± 11.8% | ND | ND |
| **AN-2**  *AN-988/40679746* |  | LBVS | 4.9 ± 8.2%  Self-fluorescence | ND | ND |
| **AN-3**  *AN-988/40679742* |  | LBVS | -4.0 ± 3.9%  Self-fluorescence | ND* | ND |
| **AS-1**  *AS-871/42110661* |  | LBVS | -1.8 ± 11.1% | ND | ND |
| **AE-1**  *AE-473/30079010* |  | PBVS | 16.0 ± 12.6% | ND | ND |
| **AH-1**  *AH-262/33733002* |  | PBVS | 26.9 ± 6.1% | ND | ND |
| **AH-2**  *AH-487/40686655* |  | PBVS | 12.1 ± 1.6% | ND | ND |
| **AG-1**  *AG-690/36720038* |  | PBVS | **83.8 ± 2.3%** | 38.2 ± 1.9% | **21.7**  **± 1.6 µM** |
| **AG-2**  *AG-205/34707042* |  | LBVS | -2.8 ± 9.7%  Self-fluorescence | ND | ND |
| **AG-3**  *AG-205/11868084* |  | LBVS | 16.3 ± 0. % | ND | ND |
| **AG-4**  *AG-670/33930027* |  | PBVS  LBVS | 5.5 ± 2.9% | ND | ND |
| **AF-1**  *AF-962/31930030* |  | PBVS | 5.2 ± 1.5% | ND | ND |

*Results are mean ± SD (n=3).

**Table S7. Pocket volumes of HDAC2, 8, 4 and 6 calculated through Connolly’s surface using CASTp server (http://sts.bioe.uic.edu/castp/).**

| **Isoform** | **PDB code** | **Pocket volume** |
| --- | --- | --- |
| **HDAC2** | 4LXZ | 191 Å^3^ |
| **HDAC8** | 1T64 (open state) | 148 (zinc pocket) + 646 (adjacent pocket) = 794 Å^3^ |
|  | 2V5X (closed state) | 145 Å^3^ |
| **HDAC4** | 4CBY | 344 Å^3^ |
| **HDAC6** | Homology model  {Butler et al. 2010} | 407 Å^3^ |

**
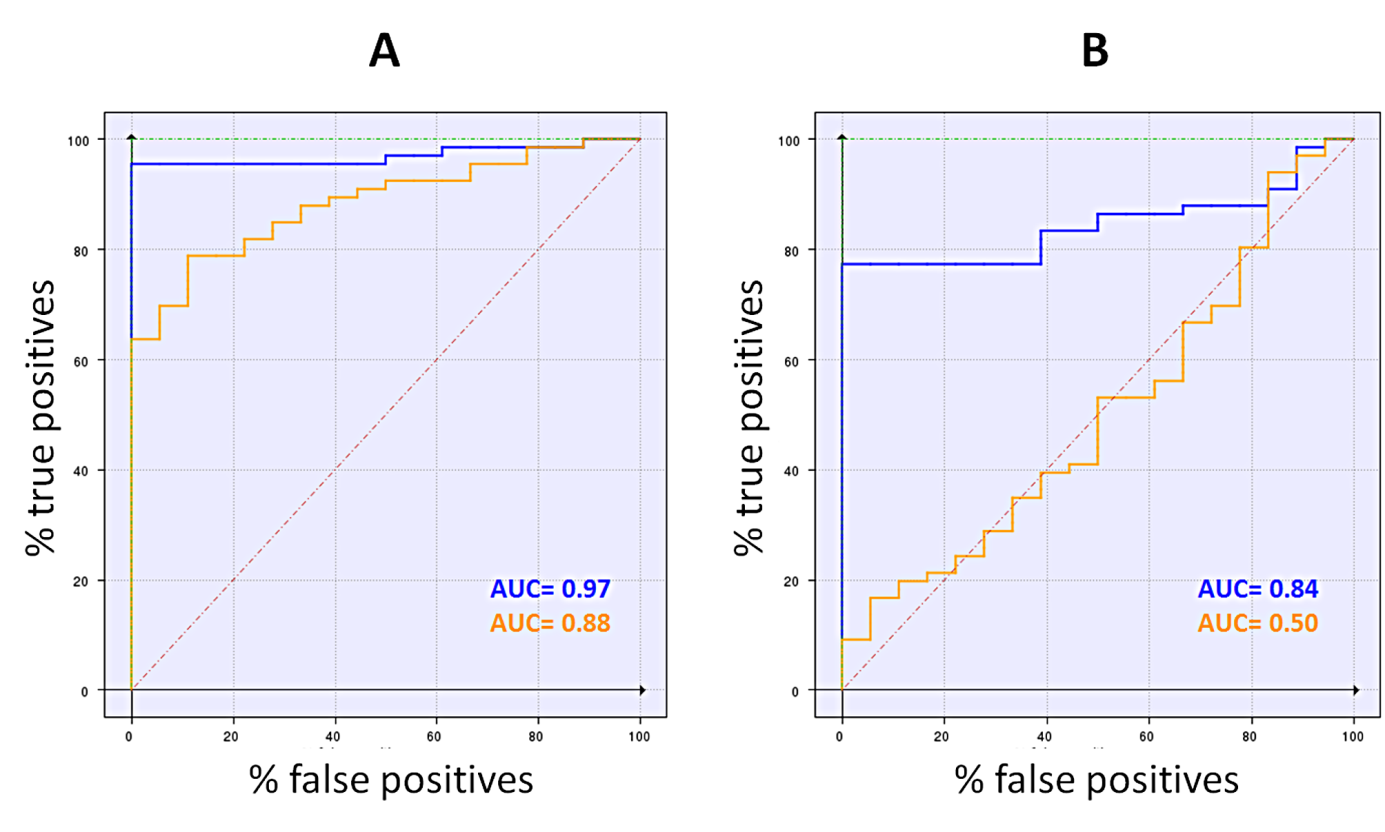
**

**Figure S1.** ROC plots for pharmacophore-based (**A**) and for ligand-based (**B**) virtual screening on the HDAC ChEMBL subset with constraints on HBD, related to N1*1 (blue) and to Glob-Prod (orange) descriptors.


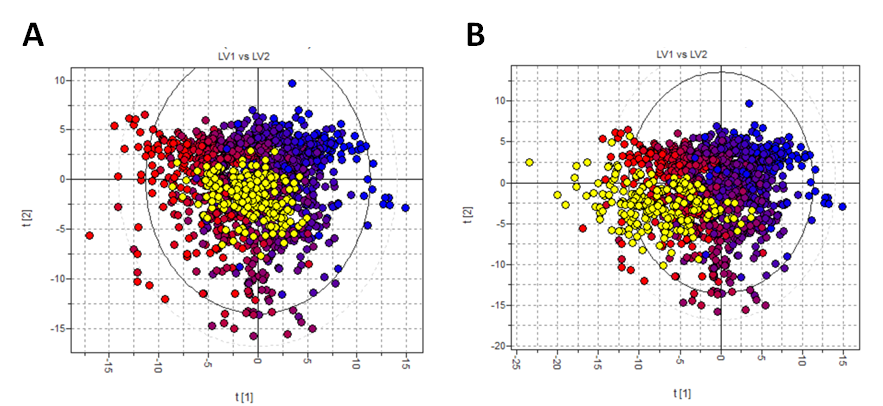


**Figure S2. (A)** Projection of the 40 virtual screening hits in the SOLY t1-t2 score plot generated with Volsurf+: blue circles are SOLY+; red circles are SOLY−; yellow circles are the projected objects. Disposition of the 8 compounds selected for biological assays are reported in black. **(B)** Projection of the 93 compounds from the HDAC ChEMBL database: blue circles are SOLY+; red circles are SOLY−; yellow circles are the projected objects.

**
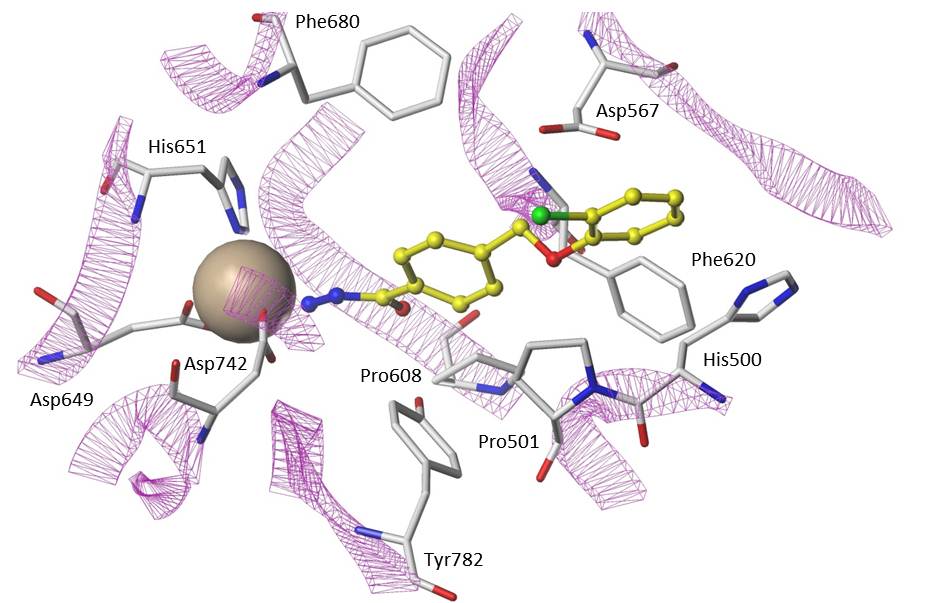
**

**Figure S3.** The best-ranked docking pose of **AK-14** (yellow ball & sticks, ChemPLP score 58.24) into the catalytic site of HDAC6 (purple ribbons). Residues involved in interactions stabilizing the complex are labeled. The zinc ion is represented as a pink ball.
